# Multimodal Emotion Recognition on RAVDESS Dataset Using Transfer Learning

**DOI:** 10.3390/s21227665

**Published:** 2021-11-18

**Authors:** Cristina Luna-Jiménez, David Griol, Zoraida Callejas, Ricardo Kleinlein, Juan M. Montero, Fernando Fernández-Martínez

**Affiliations:** 1 Grupo de Tecnología del Habla y Aprendizaje Automático (THAU Group), Information Processing and Telecommunications Center, E.T.S.I. de Telecomunicación, Universidad Politécnica de Madrid, Avda. Complutense 30, 28040 Madrid, Spain; ricardo.kleinlein@upm.es (R.K.); juanmanuel.montero@upm.es (J.M.M.); fernando.fernandezm@upm.es (F.F.-M.); 2 Department of Software Engineering, CITIC-UGR, University of Granada, Periodista Daniel Saucedo Aranda S/N, 18071 Granada, Spain; dgriol@ugr.es (D.G.); zoraida@ugr.es (Z.C.)

**Keywords:** audio–visual emotion recognition, human–computer interaction, computational paralinguistics, spatial transformers, transfer learning, speech emotion recognition, facial emotion recognition

## Abstract

Emotion Recognition is attracting the attention of the research community due to the multiple areas where it can be applied, such as in healthcare or in road safety systems. In this paper, we propose a multimodal emotion recognition system that relies on speech and facial information. For the speech-based modality, we evaluated several transfer-learning techniques, more specifically, embedding extraction and Fine-Tuning. The best accuracy results were achieved when we fine-tuned the CNN-14 of the PANNs framework, confirming that the training was more robust when it did not start from scratch and the tasks were similar. Regarding the facial emotion recognizers, we propose a framework that consists of a pre-trained Spatial Transformer Network on saliency maps and facial images followed by a bi-LSTM with an attention mechanism. The error analysis reported that the frame-based systems could present some problems when they were used directly to solve a video-based task despite the domain adaptation, which opens a new line of research to discover new ways to correct this mismatch and take advantage of the embedded knowledge of these pre-trained models. Finally, from the combination of these two modalities with a late fusion strategy, we achieved 80.08% accuracy on the RAVDESS dataset on a subject-wise 5-CV evaluation, classifying eight emotions. The results revealed that these modalities carry relevant information to detect users’ emotional state and their combination enables improvement of system performance.

## 1. Introduction

Emotions are present in almost every decision and moment of our lives. Thus, recognizing emotions awakens interest, since knowing what others feel lets us interact with them more effectively. By analyzing individuals’ behavior, it is also possible to detect a loss of trust or changes in emotions. This capability lets that specific system, such as Conversational Systems and Embodied Conversational Agents (ECAs) [1,2], react to these events and adapt their actions to improve interactions or modify the dialogue contents, tone, or facial expressions to create a better socio-affective user experience [3].

Currently, there are systems able to recognize certain emotions (or deficits) that can also help with the diagnosis of specific diseases (e.g., depressive disorders [4,5], Parkinson’s [6], etc.) and improve patients’ treatments. Another relevant application of facial expression recognition is for automotive safety. Recognizing negative emotions such as stress, anger, or fatigue is crucial to avoid traffic accidents and increase the security on the road [7] on intelligent vehicles, allowing them to respond accordingly to the driver’s state. Emotions will also play a relevant role in the future ‘Next Revolution’ that will require the development of more social robots. These robots will need to perceive people’s emotions and transmit and create their emotional states to show closer personal interactions between humans and machines [8]. Furthermore, other physiological aspects such as trust [3,9] will be interesting to define the personality of these machines.

Although, for a long time, state-of-the-art systems for speech emotion recognition (SER) had rather low accuracy and high computational cost [10,11], currently, there are new systems able to work in realtime demonstrating high performance in these conditions. Anvarjon et al. [12] proposed a lightweight CNN model with plain rectangular kernels and modified pooling layers, achieving state-of-the-art performance on the IEMOCAP (77.01% of accuracy) and EMO-DB datasets (92.02% of accuracy). The previously mentioned challenges are especially relevant when speech emotion recognition systems must be enhanced with additional multimodal input sources, such as facial expression. To address this issue, some studies such as the one developed by Franzoni et al. [13] used partial facial images employing only the mouth movements to classify emotions using transfer-learning on pre-trained models on ImageNet. Their investigation showed a loss of accuracy of only 5% on emotion detection compared to the version using the full face image on the study of four emotions: neutral, happy, surprised, and angry. Another source of information that is gaining relevance in realtime systems is textual data. Since the appearance of transformers [14] and BERT models [15], many publications have appeared in part due to the advantages of using natural language due to the reduced size of text files compared to audio or images, which requires less computing time, and the numerous datasets available to the community. As a consequence of these advantages, this modality has been applied for several tasks, for example, for sentiment and emotion recognition in several works [16,17], evidencing the capacity of this modality in multiple areas.

In this paper, we evaluate a solution to recognize emotions from multimodal information, tested on the RAVDESS dataset [18]. Speech and facial expressions are used to detect users’ emotional states. These modalities are combined employing two independent models connected by a late fusion strategy. Additionally, we introduce a subject-wise cross-validation strategy to establish a robust evaluation process to compare the contributions on the same dataset in specific and well-defined conditions to ensure its reproducibility in other scenarios and adapted to real-world conditions. On the one hand, with cross-validation (CV), the evaluation can show the statistical significance between models, not relying on the chance that the system performs well only for one or two users. On the other, the subject-wise division lets us understand its performance in real scenarios, where it is difficult to have data annotated from a new user and the system should work with the knowledge learned from other subjects.

For the speech-based and visual-based model, we evaluated two transfer-learning (TL) options: the employment of embeddings extracted from pre-trained deep neural networks; and the Fine-Tuning of these models to adapt them to our dataset for classifying emotional states.

During the study on visual modality, we discovered a mismatch between the models that process images and the ones adapted to process complete clips (or videos). When the image-type models received the individual frames of a video, they did not learn from single frames as was expected, and many of the images introduced noise to the training, making the Fine-Tuning strategies fail. This behavior may be explained by the dynamic property that emotions have and their complexity. The representation of emotions varies in intensity and passes through different states during a video, which is a very significant challenge to learn for non-temporal models.

Our models reached an accuracy of 80.08% on the ‘The Ryerson Audio-Visual Database of Emotional Speech and Song (RAVDESS)’ [18] following a subject-wise 5-CV strategy, outperforming some of the previous solutions evaluated in similar conditions. As far as we know, we have also completed the first attempt to combine speech and facial images to recognize the eight emotions in RAVDESS.

The rest of the paper is organized as follows. Section 2 describes preceding research studies related to our proposal. Section 3 summarizes the proposed methodology. Section 4 and Section 5, respectively, describe the experiments, datasets, and the main results obtained. Finally, in Section 6, we discuss the main conclusions of our study and propose future research lines.

## 2. Related Work

Emotions are considered a psychological state. Due to their interest and variety of applications, we can find studies that address this topic from different fields such as psychology, computer science, medicine, etc. [19,20,21].

The debate on emotion classification started with the study of Darwin in [22]. After this publication, many have continued along the path of studying people’s behavior to identify when the subjects exhibit a specific emotion or how their brains generate these sentiments [19].

From the psychological point of view, the literature shows that two main theories have been positioned as the focus of the discussion, among all the developed hypotheses: The first one argues that emotion can be divided into discrete categories, and on the contrary, the second one attempts to classify emotions in a more complex way using an n-dimensional space to represent them.

In the first branch of using discrete categories, Ekman [23] states that emotions can be grouped into six families: anger, fear, sadness, enjoyment, disgust, and surprise. Each of these families has sub-emotions that share common expressions that, at the same time, help to discern among the other families. Subsequent to the publications of Ekman, another of the remarkable contributions was the wheel of emotions by Plutchik [24]. His publications are a point of connection between these two visions: On one hand, the wheel contains eight discrete basic emotions; on the other hand, this wheel also has a deep dimension that represents the intensity and a dimension for measuring similarities among emotions.

Afterward, the investigation continued until the apparition of the n-dimensional theories as ‘the circumplex model of affect’ of Posner and Rusell in 2005 [25], which establishes that emotions arise as a product of two independent neural systems, one that informs the valence (i.e., how pleasant or unpleasant an emotion is for a person) and another that represents the arousal (i.e., how intense or soft it is). This new theory seeks to incorporate some experimental results obtained in neuroscience.

These psychological theories are the base from which most of the currently available datasets used by the scientific community for emotion recognition were created; there are some discrepancies in the labels of the available datasets [18,26,27,28,29,30]. Depending on the corpus, emotions are tagged in terms of a group of families, following Ekman [18,27]; in terms of valence and arousal [30], as Posner and Rusell suggested; or following other approaches [26,28,29]. These datasets have promoted the investigation of new features and models for automatic emotion recognition.

Far from being a closed field of study, there are still many researchers investigating emotion modeling, such as the recent works by Furey et al. [31] and Franzoni et al. [32]. In [31], Furey and Blue propose to introduce and test new temporal indicators of emotional states such as hobbies, habits, sleep time, physical activity, etc., as complementary sources to speech, text, or facial expressions. In [32], Franzoni et al. explain how the tracking of emotions may be possible using common devices, such as smartphones or cameras, to improve human–machine communications in emergency scenarios where having emotional and contextual information could help to solve more efficiently this type of situations that require a quick interaction between different agents.

### 2.1. Speech Emotion Recognition

When people engage in natural conversational interactions, they convey much more than just the meanings of the words spoken. Their speech also transmits their emotional state and aspects of their personality [10,33]. Paralanguage refers to properties of the speech signal that can be used, either consciously or subconsciously, to modify meaning and communicate emotion. Examples of paralinguistic features include those that accompany and overlay the contents of an utterance and change its connotation, such as pitch, speech rate, voice quality, and loudness, as well as other vocal behaviors, such as sighs, gasps, and laughter. Paralinguistic properties of speech are very important in human conversation as they can affect how a listener perceives an utterance. Schuller and Batliner [11] presented a detailed survey of computational approaches to paralinguistics, examining the main methods, tools, and techniques used to automatically recognize affect, emotion, and personality in human speech. Berkeham and Oguz have very recently presented an extended survey of emotional models, databases, features, preprocessing methods, supporting modalities, and classifiers for speech emotion recognition [34].

For many years, the most common approach for emotion recognition was to apply feature engineering from hand-crafted features to detect those that most contribute to identifying emotions. An example of this line of investigation is the work of Ancilin and Milton [35] that evaluated a method for the extraction of Mel frequency cepstral coefficients based on the magnitude spectrum instead of the energy spectrum and exclusion of discrete cosine to transform and extract the Mel Frequency Magnitude Coefficient. With time and to reduce the researchers’ efforts, some frameworks emerged to obtain the most successful features automatically, such as OpenSmile [36] or Praat [37].

Today, the trend is evolving to the use of certain well-known features as MFCCs with simple models, as in the work of Bhavan et al. [38]. They use MFCCs with spectral centroids as input features, introduced into a bagged ensemble of Support Vector Machines, achieving an overall accuracy of 72.91% for RAVDESS.

However, there has also been the use of Deep Learning models capable of processing hand-crafted features or a complete audio record. For example, Singh et al. [39] proposed the use of hand-crafted features to feed deep neural networks. Prosody, spectral, and voice quality-based features were used to train a hierarchical DNN classifier, achieving an accuracy of 81.2% on the RAVDESS dataset. Pepino et al. [40] combined hand-crafted features and deep models using eGeMAPS features together with the embeddings extracted from Wav2Vec to train a CNN model. They achieved an accuracy of 77.5% applying a global normalization on this dataset. Issa et al. [41] also proposed a new method for feature extraction calculating Mel-frequency cepstral coefficients, chromagram, Mel-scale spectrogram, Tonnetz representation, and spectral contrast features from speech files. These features were the inputs of a one-dimensional CNN. Their proposal obtained an accuracy of 71.61% for RAVDESS. Other works as proposed in [42,43,44], also employed CNNs, MLPs, or LSTMs to solve the emotion recognition task on RAVDESS using spectrograms or preprocessed features to feed these models, obtaining accuracies of 80.00%, 96.18%, and 81%, respectively.

Concerning the research line based on exploiting the advantages of using deep learning models, many publications have also employed Transfer learning techniques by extracting embeddings or fine-tuning pre-trained models [45,46,47] rather than training the models from scratch, as they did in most of the previously presented publications. Some of the most influential and recent libraries for solving audio tasks are DeepSpectrum [48], and PANNs [49]. Both libraries allow for extracting embeddings from their pre-trained models, but PANNs also let us retrain their models.

A major concern of the investigation of this paper is that despite the increasing number of publications that use RAVDESS, they lack a common evaluation framework, which makes it difficult to compare contributions. For example, Atila et al. [43] achieved 96.1% accuracy, and although they used a 10-CV evaluation, they did not specify how they distributed the users in their folds, not being clear whether in each fold the same user takes part of the training and the test set or not, which is crucial information to replicate their setup for comparing proposals. Another example of a different setup appears in Pepino et al. [40], where they used 20 users for training, two for validation, and two for the test set, classifying only seven of the eight emotions that the dataset has. In these conditions, models’ performance would be subject to two single actors’ evaluation, which may not reflect the real-world representation of other individuals. For this reason, we consider it necessary to establish a common evaluation design that we introduce and explain along with the paper that consists of a subject-wise 5-CV strategy using the eight emotions recorded in the RAVDESS dataset.

In addition to the proposed setup, the contribution of this paper is in the line of using deep-learning models rather than studying new hand-crafted features. Specifically, we compare the performance of two CNN-type pre-trained models that exploit spectrogram representation to extract features. These models belong to the DeepSpectrum and PANNs libraries employed previously for solving similar audio-based tasks [50,51,52,53], which makes them suitable for speech emotion recognition too. To our knowledge, they have not been compared in any other investigation, using two TL techniques (Feature Extraction and Fine-Tuning).

### 2.2. Facial Emotion Recognition

Although vocal information is an essential modality for predicting emotions, the results of the emotion recognizer could improve by incorporating other modalities, as demonstrated by Singh et al. [39], where incorporating textual features enhanced the results of the speech emotion recognizer. In our case, we include the information that facial expressions give for emotion recognition.

As happens with SER, some tools can extract information of the facial morphology and detect expression variations between frames of a video. An example of these tools is the dlib library [54] that let us estimate a landmark position on a face. Landmarks consist of the coordinates of some key points allocated around the eyebrows, eyes, nose, mouth, and jaw, which describe the expression of a person at that moment. Nguyen et al. [55] demonstrate that landmarks encapsulate meaningful information about the facial expression of a person. Another example of a feature related to emotions is the Action Units (ACs). Action Units are an evolved version of landmarks that reflect facial movements and not just positions of specific parts of the face. Bagheri et al. [56] extracted AUs and introduced them into a seven-layer auto-encoder, recognizing emotions with a high accuracy rate.

Despite the success of these features, the results in emotion recognition have improved with the appearance of new Convolutional Neural Network (CNN) models that receive the whole image at their input. EmotionalDAN [57] is one example of these models, which can solve emotion, valence, and landmarks recognition at once. Their results confirm that the three tasks are associated since introducing the landmark recognition branch guides the training of the weights for emotion recognition and improves the final accuracy. However, CNNs lack an attention mechanism that can identify the most appropriate parts of an image from which to learn. Spatial Transformer Networks (STN) [58] aim to detect the principal regions that appear on an image and correct spatial variations by transforming the input data, as happens in Deep-Emotion [59]. Deep-Emotion uses an STN architecture to address emotion recognition, emphasizing that these models are appropriate to solve this task. For this reason, we employed this type of architecture to solve facial emotion recognition too. Specifically, we utilized the guided STN pre-trained model on AffectNet for valence recognition using saliency masks from Luna-Jiménez et al. [60] and adapted it to solve facial emotion recognition.

Since our target was to estimate emotion in videos and not just in frames (or images), we evaluated two strategies to give a final prediction on the whole video, collapsing the outputs at the frame level returned by the STN. The first one used max-voting over the predictions of the frames, assigning the most frequent class to the video. The second strategy introduced a temporal model, concretely, a bi-LSTM with attention to extracting the video verdict from the embeddings derived from the STN or the posteriors of each frame.

### 2.3. Multimodal Emotion Recognition

Among the techniques that appear in the literature to merge modalities, two main methods stand out: early fusion or late fusion.

Early fusion signifies the concatenation of features from several sources or modalities extracted from different pre-trained models. These features are joined before training a final model. In contrast, late fusion consists of a first stage to train as many models as modalities; and a second stage to create a final model that learns from the posteriors or the concatenated features calculated in the first stage. Sometimes the frontier between these two techniques is not too evident since fusion strategies could happen at any moment during the training. For this reason, in the literature, we can also find some work concerning hybrid fusions [61].

One advantage of early fusion is that it can detect correlations between features to remove redundant information and learn the interaction between different modalities. However, it may encounter synchronization problems when aligning data from several modalities due to the different sampling rates, and it also may suffer problems when the joined embeddings reach high dimensions [62,63]. Some works such as the one proposed by Deng et al. [64] are included in this method. In their investigation, they extracted embeddings from two modalities, text and audio. For the audio component, they used fine-tuned networks (VGG, YAMNET, and TRILL) to obtain the representative features associated with the recording. Then, they applied the same procedure for the textual component using the T5 transformer. Finally, the embeddings were concatenated and passed to a transformer model with co-attention that enhance the most relevant slots of each embedding to create a fused representation, used posteriorly to train a final classifier. The fusion of these two modalities boosted the accuracy of the emotion recognizer in two datasets, IEMOCAP and SAVEEE.

As an alternative to an early fusion strategy, we have the fusion at the decision level or late fusion. Sun et al. [62] applied a late fusion strategy using a bi-LSTM to combine the predictions of the models per modality. More specifically, they trained a bi-LSTM model with an attention layer per one of their three used modalities (audio, video, and text) to recognize arousal and valence utilizing features extracted from pre-trained models on other tasks. Then, they combined the posteriors of the bi-LSTM models following a late fusion strategy to learn the final LSTM model.

Due to the simplifications in terms of synchronization that the late fusion strategy offers and its good performance in some previous works on similar tasks [62,65], we decided to apply a combination of the posteriors of each trained model on one of the two modalities (aural or visual) and then, feed a final model with them.

To conclude, although, in the literature, other works also perform multimodal emotion recognition on RAVDESS, such as Wang et al. [66], that used facial images to generate spectrograms, which were then used as data augmentation to improve the SER model performance in six emotions; to our knowledge, our work is the first that evaluates a late fusion strategy using the visual information of RAVDESS for facial emotion recognition using the eight emotions of the dataset with a pre-trained STN and the aural modality.

## 3. Methodology

Our proposed framework for multimodal emotion recognition consisted of two systems: the speech emotion recognizer and the facial emotion recognizer. We combined the results of these subsystems with a late fusion strategy.

**Figure 1:**
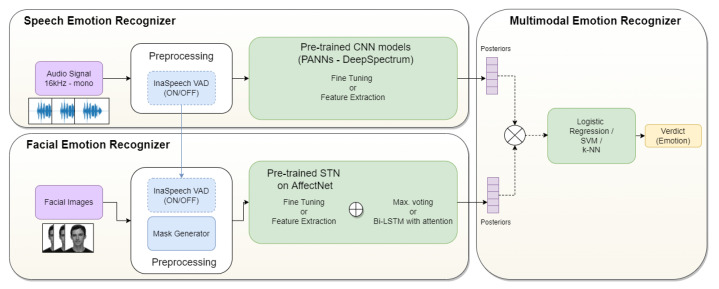
Block diagram of the implemented systems.

Figure 1 shows a block diagram with the main modules of our systems and a summary of the compared TL strategies (Fine-Tuning and Feature Extraction); the comparison of several models, such as those implemented in the PANNs and DeepSpectrum libraries; or the application of max. voting or a bi-LSTM in the facial emotion recognizer.

In this section, we describe in detail the used dataset, each subsystem, and the strategies applied to develop them.

### 3.1. The Dataset and Evaluation

In this study, we used the RAVDESS dataset [18], mainly because it is a free of charge reference corpus for the scientific community for speech emotion recognition [39,67,68], but also because of its suitability for our experiments.

The Ryerson Audio-Visual Database of Emotional Speech and Song (RAVDESS) contains 7356 recordings with acted-emotional content. These files are divided into three modalities (full AV, video-only, and audio-only) and two vocal channels (speech and song). Each file contains a single actor representing an emotion that could be one of the eight following categories: calm, neutral, happy, sad, angry, fearful, surprised, and disgusted. These expressions are produced at two levels of emotional intensities (regular and strong) except for the neutral emotion that only contains regular intensity. In our study, we maintained these eight emotions to respect the conceptualization of the task when the researcher acquired the dataset against other works that recognize only a subset of these emotions.

For our experiments, we only used the full AV material and the speech channel since we focused on the task of audio–visual emotion recognition in speech but not in songs. This selection reduced the number of files to 1440 videos that had a maximum and minimum duration of 5.31 and 2.99 s, respectively. The videos belonged to 24 gender-balanced actors, vocalizing only two lexically-matched statements in a neutral North American accent, making it suitable to study the paralinguistics associated with emotions, isolating the lexical and reducing the bias in emotional expressions that culture may induce. Among its advantages, it had a balanced number of files per emotion, which avoided problems derived from training algorithms with non-balanced data.

Despite its simplifications, this dataset still presents important challenges for emotion recognition. A proof of this is the human accuracy rate reached: 67% using only speech stimuli and 75% using visual information.

We evaluated our results following a subject-wise 5-fold cross-validation strategy. The division in folds was random and stratified in terms of classes and users, i.e., each fold had a similar number of samples per class randomly selected, but we always maintained each actor in the training or the validation sets, never in both.

The distribution of the actors on the validation set in each fold was as follows:

- Fold 0: (2, 5, 14, 15, 16)
- Fold 1: (3, 6, 7, 13, 18)
- Fold 2: (10, 11, 12, 19, 20)
- Fold 3: (8, 17, 21, 23, 24)
- Fold 4: (1, 4, 9, 22)

We developed this setup following the proposal by Issa et al. [41], which applied a similar cross-validation methodology independent of the users and using the eight classes of the dataset, with the difference that they did not specify the actors used in each fold, and we did. This evaluation procedure enables comparing our contribution with the speech modality of this previous work. In the results section, we report the average accuracy achieved by the folds at the video level. We also include a confidence interval to evaluate the significance of our methods and compare scenarios.

In addition to the confidence interval, we performed a nonparametric study using the Cochran’s Q test to determine whether there were differences in the success rate between the top models of the three modalities. As we found significance, we applied a McNemar’s test to discover which model was the best in terms of accuracy. The Bonferroni method was used to perform pairwise comparisons following a significant overall test result.

### 3.2. Speech Based Emotion Recognizer

**Figure 2:**
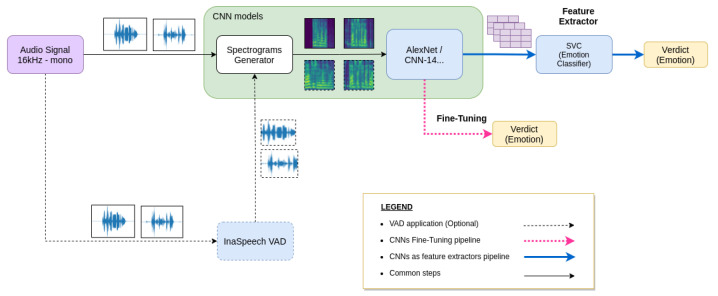
Proposed pipelines for speech emotion recognition.

Training a Convolutional Neural Network (CNN) from scratch for emotion recognition needs a high amount of data to discern between different classes. Transfer learning techniques can alleviate this load by customizing pre-trained models in two different ways: Feature Extraction and Fine-Tuning. In this section, we detail how we employed these two techniques to train our speech emotion recognizer. Figure 2 shows the whole pipeline.

#### 3.2.1. Feature Extraction

In this case, the idea was to reuse a pre-trained speech recognition network to extract meaningful features from the samples of RAVDESS. These features may be specific to the original classification task though still useful for representing the content of the RAVDESS recordings. Then, we simply added a new classifier, which was trained from scratch, on top of the pre-trained model so that we could repurpose the feature maps learned previously for the RAVDESS dataset (only the final classification part was trained and adapted).

For this method, we used two different frameworks: DeepSpectrum [48] and PANNs [49].

These frameworks allow reuse of pre-trained CNNs to extract embeddings. Their implemented models receive speech signals. Then, these signals are internally processed and converted into spectrograms. Once the spectrograms are available, the networks learn the new task (speech emotion recognition) from this frequency-based format.

The main difference between both frameworks is that DeepSpectrum contains models pre-trained on the ImageNet dataset [69], a large dataset for object recognition; whereas PANNs have models trained on the large-scale AudioSet dataset. Unlike ImageNet, AudioSet is a corpus with million of audio events, specifically designed to classify sounds. Therefore, the models trained on PANNs are closer to sound identification tasks than those networks included in DeepSpectrum.

For both frameworks, we subsampled the audios to 16 kHz and converted them into monochannels using the FFmpeg tool [70]. We applied this subsample because, in the original paper [49], the CNN-14 model reached its top performance using this frequency and also to use the pre-trained models of the frameworks that work at this frequency.

After the preprocessing stage, they passed to the frameworks that internally create a frequency-based representation of the recordings.

From DeepSpectrum, we decided to extract the embeddings from the last layer of the AlexNet architecture (fc7), as proposed by Amiriparian et al. [48]. This layer returns a 4096-dimensional vector per record.

Regarding PANNs, we used the CNN-14 model to extract embeddings from its last layer. This layer has a dimension of 2048, approximately half of the size of the AlexNet embeddings.

Once they were computed, we used these embeddings to train a speech emotion recognizer with a Support Vector Machine (SVM) implemented in sklearn [71] with an ‘RBF’ kernel and a regularization parameter of C=0.001. Figure 2 shows this pipeline following the black and the blue arrows.

#### 3.2.2. Fine-Tuning

As an alternative to the embeddings and to reuse the previous networks’ knowledge, we also fine-tuned the pre-trained models. By Fine-Tuning a base pre-trained model, we unfroze a few of its top layers and jointly trained both the newly-added classifier layers and the last layers of the base model. This technique allowed us to “fine-tune” the higher-order feature representations in the base model to adapt them to the new specific task.

Notice that both networks, AlexNet and CNN-14, apart from the base task, also differ in the total number of parameters since AlexNet contains fewer weights than CNN-14. These differences in sizes, as well as the images used for training both networks, may explain differences in results between both CNNs. Figure 2 shows this pipeline following the black and the dotted pink arrows.

As the silences may not be relevant to emotion recognition, as described in [72], we did some experiments with a Voice Activity Detector (VAD) to reduce the processing material.

The chosen VAD was the InaSpeech VAD [73] because of its performance in complex scenarios. For the version with VAD, we maintained the regions between the first moment that speech was detected until the last instant predicted as speech to preserve the same number of samples in the dataset, obtaining a single chunk per record. In contrast, for the version without VAD, we preserved the whole original record, switching off the VAD.

The VAD distinguished between ‘speech’, ‘noise’, ‘noEnergy’, and ’music’. For the recordings where the detector did not detect any speech regions, we maintained the original audio.

Figure 2 also represents the scenario with the VAD following the black dotted arrows instead of the solid black line.

### 3.3. Facial Emotion Recognizer

For the facial emotion recognizer, firstly, we extracted the frames of the videos at 30 fps and removed white lateral pixels to convert them into squared images of 720 × 720 from their original size of 1280 × 720 to later resize them to 48 × 48, the size that the pre-trained model (an STN) expects at its input. With the removal of the lateral pixels, we avoided deformations that may cause rate degradation due to changes in the aspect ratio of the samples.

We also extracted saliency maps from the original frames, since the STN receives tuples, the original image, and its associated saliency map. To generate the visual saliency maps, we employed the pre-trained CNN of Kroner et al. [74], on the salicon dataset [75]. The reason for choosing this network was due to its size–performance balance. This model was lighter than others but achieved as good metrics as some state-of-the-art saliency predictors.

Once we generated the frames and saliency maps of the videos, they were used as single samples that heritaged the label of its parent video, since the STN was trained in a supervised way from single images and did not accept sequences. As we did with the audios, we compared the performance of applying Feature-Extraction and Fine-Tuning, but this time with a pre-trained STN for Facial Emotion Recognition.

In both procedures, we considered a static solution using a pooling (max. voting) and a dynamic solution using a bi-LSTM with an attention layer. These strategies aimed to address the video emotion recognition problem from the predictions at the frame level. In this section, we detail the differences between these approaches.

#### 3.3.1. Feature Extraction

The pre-trained facial emotion recognition model was a modified STN that used saliency maps to capture the principal regions of a face, since the performance of these models improved if the STN had access to more processed information about the relevant figures and shapes that appear in an image, as suggested in the previous work of Luna-Jiménez et al. [60].

**Figure 3:**
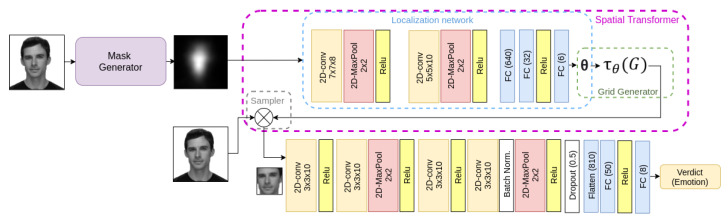
Spatial Transformer CNN architecture with visual saliency-based masks.

The STN model was similar to the model shown in Figure 3. This model was trained on the AffecNet dataset [26] and it received the facial images whose emotion has to be classified. Then, the Mask Generator created a saliency-based mask for each frame that passed to the localization network. The localization network learned the transformation parameters from this image and sended them to the sampler. The sampler received the θ parameters and the original image as inputs and returned the transformed version of the original image. This transformed image was passed to the second CNN to solve the facial emotion recognition problem.

On top of the STN, we attached a new model to compact its outputs and give a final prediction at the video level. We tested two possible models: a feature pooling strategy and a sequential model. The first one applied the max. voting algorithm over the individual frames to extract the most repeated class and assign it to the video, whereas the second strategy relied on a sequential model to predict the video verdict from the embeddings generated by the STN from each frame.

To generate one of our pre-trained models and apply the ***Simple feature pooling strategy***, we trained the STN recognizing emotional classes on AffectNet, seven in total.

In the first proposed method, the FER model was applied frame by frame, thus producing as many classification results as frames from the video. Then, the class of the input video was determined by a majority vote of the resulting frames’ predicted labels.

This voting strategy, adopted as our baseline, had two main benefits: the first one was its simplicity, and the second was the average effect. For example, when an input video had a large deviation from a prototype emotion locally at several frames, these frames did not severely influence the final recognition result; they were treated just as several incorrect votes, and thus, they were neglected successfully through the majority vote.

On the other hand, this method had an evident drawback: similar to speech recognition and sentence classification, video-based facial expression and emotion recognition involves analyzing sequential data that may exhibit a natural temporal ordering; however, this heuristic aggregation of individual frame-level predictions ignored the underlying temporal order of the input sequence frames, which in turn may result in suboptimal discriminative capability.

As an alternative to the majority voting strategy, we adopted a ***Sequential Model***, which was expected to be particularly powerful for sequential data.

For the sequential model, we employed an RNN on the posteriors of the seven-class pre-trained STN on AffectNet and the embeddings of the three-class pre-trained STN for valence recognition (positive, neutral, and negative), respectively.

This RNN uses a Long Short-Term Memory (LSTM) network. These models can process their inputs sequentially, performing the same operation, $h_{t}=f_{W}\left(x_{t},h_{t-1}\right)$, on each of the different elements that constituted our input sequence, where $h_{t}$ is the hidden state, *t* the time step, and *W* the weights of the network.

As input to this model, we introduced the embeddings or posteriors generated by the STNs for each frame of the video ($x_{1},x_{2},\ldots x_{N}$), to learn the temporal relations from the spatial similarities discovered by the STN and give a final prediction at the video level.

**Figure 4:**
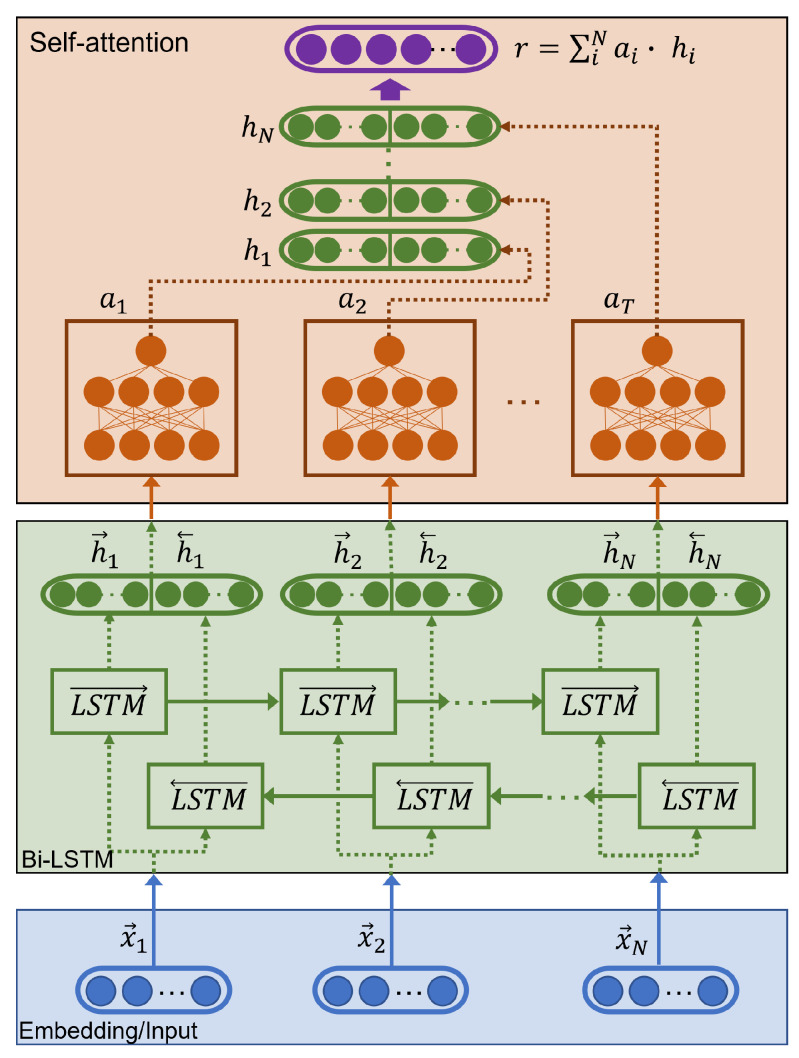
Bidirectional-LSTM with attention mechanism for facial emotion recognition at the video level. Modified version from source [77].

Regarding the architecture, our temporal model consisted of one or more bidirectional-LSTM layers with a deep self-attention mechanism as proposed by Baziotis et al. [76]. In Figure 4, we show the structure of the bi-LSTM.

Each bi-LSTM layer behaves in a bidirectional way, which allowed us to collect the information of the sequence in both directions from the hidden states $h_{1},h_{2}\ldots ,h_{N}$ of the LSTMs. In particular, a Bi-LSTM consists of two LSTMs, *forward*$\vec{LSTM}$ that allows the analysis of the frames from $x_{1}$ to $x_{N}$, and an *inverse or backward*$\overset{\leftarrow }{LSTM}$ that allows a similar analysis to be carried out but in the opposite direction, from $x_{N}$ to $x_{1}$.

To obtain the emotional tag from our bi-LSTM layer, we concatenated for each embedding of a frame the outputs obtained from the analysis performed in each specific direction (see Equation (1) in which || corresponds to the concatenation operator and *L* to the size of each LSTM).

(1) $$h_{i}=\vec{h_{i}}\left|\right|\overset{\leftarrow }{h_{i}},whereh_{i}\in R^{2L}$$

The attention layer identified the most informative frames when determining the emotion of the video. The actual contribution of each embedding was estimated through a multilayer perceptron (MLP) with a nonlinear activation function (tanh), similar to that proposed by Pavlopoulos et al. [78].

The attention function *g* is a probability distribution on the hidden states $h_{i}$, that allowed us to obtain the attention weights $a_{i}$ that each embedding (or frame of the video) received. As the output of the attention layer, the model computed the linear combination of the LSTM outputs $h_{i}$ with the weights $a_{i}$.

Finally, we used *r* as a feature vector that we fed to a final task-specific layer for classification. Specifically, we used a fully connected layer, followed by a *softmax* operation, which output the probability distribution over the classes.

#### 3.3.2. Fine-Tuning

As a comparative strategy to the features extraction and, as we did with the speech emotion recognizer, we fine-tuned the STN with the samples extracted from the RAVDESS dataset.

We changed the last three-neuron fully connected layer of the AffectNet pre-trained STN, used for sentiment recognition of three categories: ‘Negative’, ‘Neutral’, and ‘Positive’ to an eight-neuron fully connected layer to solve the new problem of emotion recognition and to fit the eight emotions that RAVDESS annotated. Later, we retrained the whole network with the frames that conformed to the RAVDESS dataset, as Figure 3 shows.

As we will see in Section 5, AffectNet weights were used as initialization to improve the learning of the network, since using the weights learned on sentiment recognition was more beneficial for the training than random weights initialization for emotion recognition. However, these weights still needed to be adapted, since there are differences between sentiment recognition and emotions recognition; for this reason, we performed Fine-Tuning on the RAVDESS dataset.

Again, we evaluated the static and sequential strategies using the max-voting algorithm and the bi-LSTM with an attention mechanism. This time, we extracted the posteriors and embeddings from the fine-tuned model. To apply the pooling-based strategy, we extracted the posteriors estimated in the last fully connected layer to calculate the rates at the video level.

Unlike the max-voting strategy, the sequential model can receive other inputs different from the posteriors. For this reason, we compared the performance of the bi-LSTM when it was fed with: the posteriors, the embeddings extracted at the next-to-last layer of 50 neurons (fc50), or the embeddings from the third to last layer (an 810-dimensional flattened layer) of the STN.

### 3.4. Multimodal Recognizer

There are several techniques to combine information from different sources, although two of the most popular are early fusion and late fusion.

In our case, we chose to merge information at the decision level (late fusion) to avoid synchronization issues, since the audio models worked with the whole audio sequence, whereas the STN of the FER model was trained using single frames. Hence, an early fusion would require summarizing the features of the STN with a temporal model or a pooling strategy before concatenating them with the aural embeddings. Moreover, from the analysis of the feature extraction techniques, we will see in Section 5 that the results without applying Fine-Tuning were significantly lower, and we considered that an early fusion strategy may cause suboptimal performances. For these reasons, we implemented a late fusion. First, we extracted the posteriors from the eight-neuron layer of the top Speech Emotion Recognizer, the fine-tuned CNN-14 on the RAVDESS dataset, and the posteriors of the bi-LSTM trained with the outputs of the STN for visual modality. Then, we concatenated these embeddings to introduce them into the model used in the late fusion. To apply the fusion, we tested several models varying certain hyperparameters of these models that will be explained in Section 4. For this final model, we compared the performance of a Logistic Regression, a k-NN with max. voting, and an SVM with ‘linear’ and ‘rbf’ kernels, implemented using Python and the sklearn library [71].

From the experiments, the best results were reached using linear models.

## 4. Experiments

In this section, we describe the setup per modality: the training parameters (batch size, epochs, optimizers, learning rate, etc.) and the differences between the tested architectures (the type of models, the number of layers, and neurons). Section 4.1 describes the configuration for the two TL strategies applied on the Speech Emotion Recognizer: Feature Extraction and Fine-Tuning. Section 4.2 presents the Feature Extraction and the Fine-Tuning parameters used in the Facial Emotion Recognizer.

### 4.1. Speech Emotion Recognizer Setup

As we commented in Section 3.2, for the Feature Extraction, we selected the 4096-dimensional embeddings from the fc7 of the AlexNet network from DeepSpectrum and the 2048-dimensional embeddings from the last layer of the CNN-14 of the PANNs library. Then, these embeddings were introduced into the SVM to classify the emotions of the RAVDESS dataset.

Concerning the training configuration and hyperparameters, for the Fine-Tuning experiments of the CNN-14 over the learned weights on AudioSet, we used a batch size of 24 samples and a maximum number of training iterations of 30,000.

As we were solving a classification task, we utilized the negative log-likelihood loss implemented in PANNs.

To optimize this objective function, we employed the default optimizer of the library, Adam, with a learning rate of 0.001, betas with values 0.9 and 0.999, and epsilon of $10^{-8}$.

For the AlexNet network, we used the cross-entropy loss with an SGD optimizer, a learning rate of 0.001, a momentum of 0.9, and a weight decay of 0.0005. We trained the network during 500 epochs. We also tested the same loss and optimizer as the CNN-14; however, as the network did not learn, we changed to the original loss and optimizer used for pre-training the model on ImageNet.

### 4.2. Facial Emotion Recognizer Setup

Continuing with the visual experiments, for the Feature Extraction, we compared the performance reached by using the embeddings from the 810-dimensional flattened layer, the 50-dimensional fully connected, and the posteriors of the STNs trained on AffectNet for sentiment or emotion recognition.

Regarding the attached models on top of the STN, for the max-voting algorithm in the Feature-Extraction version, we had to assimilate the same posterior for the ‘Calm’ and ‘Neutral’ classes since AffectNet did not contain the ‘Calm’ label. For the rest of the results with the bi-LSTM, we used eight emotions.

For the training of the sequential model, we chose a batch size of 64 with a learning rate of 0.001. To avoid overfitting, we also implemented an Early-Stopping strategy to finish the training when the F1 score did not improve in 30 epochs. Regarding regularization, both the bi-LSTM and the attention layers had a 0.3 dropout rate.

As a cost function, we utilized the weighted cross-entropy loss implemented in PyTorch [79], with an Adam optimizer and a learning rate of 0.001. The same configuration was used for the model trained on AffectNet.

The encoder layers varied in length and depth depending on the size of the input embeddings:

- For the training of the bi-LSTM with the embeddings extracted from the flattened layer of size 810, we ran experiments with two identical bi-LSTM cells of 50, 150, 200, or 300 neurons and two attention layers.
- For the version with the 50-dimensional embeddings, we tested one or two stacked bi-LSTM layers with 25, 50, or 150 neurons. The number of layers of the attention mechanism was the same as the number of stacked bi-LSTM layers.
- For the posteriors of the STN, we trained six models modifying the number of bi-LSTM layers in one or two and the number of neurons in the range [25, 50, 150]. When we used two layers, both layers were identical. For each experiment, the number of layers of the attention mechanism coincided with the number of layers of the bi-LSTM.

For the Fine-Tuning of the STNs on RAVDESS, we changed the last fully connected layer with an eight-neuron layer, according to the number of classes to predict. For the training, we selected a batch size of 128 samples and a maximum number of training epochs of 500. We applied the same Early-Stopping strategy as in the STNs, checking the validation accuracy instead of the F1 score, and the same loss and optimizer. Regarding regularization, we only used dropout with a 0.5 probability.

In this case, the max-voting algorithm stacked at the top of the STN used the posteriors of the last eight neuron-layer of the fine-tuned STN. Regarding the sequential model, we tested the same combination of layers and dimensions commented on in this section for the feature-extraction version.

We repeated these experiments for the version with VAD and without VAD and in Section 5 we reports the models that achieved the highest accuracy on the task.

### Multimodal Emotion Recognizer Setup

For the late fusion models, we studied an SVM, a Logistic Regression, and k-NN algorithms varying the values of some of their hyperparameters.

For the SVM, we compared two types of kernels, a ‘linear’ and an ‘rbf’ kernel, varying the *C* parameters in steps multiples of 10, starting at 0.0001 until reaching a *C* of 100.

For the Logistic Regression, we also varied the regularization parameter (*C*) in steps multiples of 10 in the range from 0.1 to $10^{6}$.

Finally, for the k-NN, we tested with 10, 20, 30, 40, and 50 as the number of neighbors.

In all the experiments, we stopped the grid search when the performance started to decrease or saturate. In Table A3, the reader can see the tests performed and the results obtained for the different strategies applying VAD or not in each modality. Notice that the results and experiments were only performed and reported on the best models found during the Fine-Tuning for the two modalities, aural and visual.

## 5. Results

In this section, we summarize the main results obtained in our proposals for speech, facial, and multimodal emotion recognition. We also include an analysis of the errors and a comparison of our work with related proposals evaluated on the same dataset.

### 5.1. Speech Emotion Recognition Results

Table 1 summarises the performance of the Speech Emotion Recognition models tested. For the Feature Extraction strategy on AlexNet and CNN-14, the results suggested that AlexNet’s embeddings outperformed the accuracy reached by CNN-14’s embeddings when they were passed to an SVC. This difference may be explained by the dimension of the embeddings, where AlexNet embeddings have a size of 4096, the embeddings extracted from CNN-14 have a dimension of 2048, half of the size.

However, for the Fine-Tuning strategy, we observe that the tendency changes. The CNN-14 had the best accuracy after Fine-Tuning it on RAVDESS using the whole recordings as input to the model. It outperformed AlexNet results by 15.86% in the same conditions, without using VAD. One cause of this difference could be the nature of the training data, since AlexNet had pre-trained weights learned from images of ImageNet, whereas CNN-14 was trained using Mel spectrograms extracted from audios. Hence, the second architecture seems more adapted to the task. Another limitation of AlexNet regarding CNN-14 was the number of parameters. Unlike CNN-14 with 81M parameters, AlexNet only had 61M. This fact could also help explain the maximum accuracy that each network could reach with such a complex problem as emotion recognition.

When comparing the two TL strategies, Feature Extraction against Fine-Tuning, we can see that adapting the model to the task achieved a performance improvement against using only the embeddings from another task. This result suggests that our dataset had enough size to let the networks learn effectively from the recordings, and that the knowledge embedded in the pre-trained weights was robust and compatible with speech emotion recognition.

Regarding the inclusion of the InaSpeech VAD, the results did not show that it improved the learning of the task since in no case, did they change in the version with VAD. The experiments suggest that removing the initial and final silences does not disrupt the final performance of the models.

**Figure 5:**
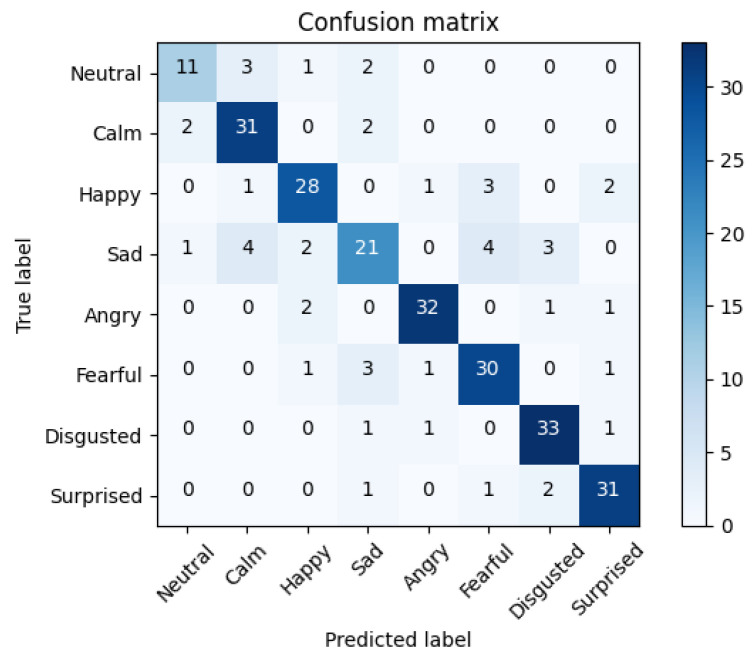
Average confusion matrix of the fine-tuned CNN-14 experiment with an accuracy of 76.58%.

To understand the errors of the top solution, we extracted the confusion matrix of the fine-tuned CNN-14 experiment with an accuracy of 76.58%. The confusion matrix displayed in Figure 5 is the rounded average value of the errors and the correct predictions obtained from the folds of the 5-CV. This matrix will display an average of 288 samples (1440/5).

The Figure reveals that the network showed a good performance except for some samples. The ‘Sad’ class contained the highest number of errors, mistaking this class in most cases with other emotions such as ‘Disgusted’ or ‘Fearful’, although it also confused this emotion with ‘Calm’, which may be occasioned by the low arousal level of both emotions.

From the result of the speech modality, we can confirm that Fine-Tuning pre-trained models on similar tasks helped reach higher scores than using the embeddings of the models without adaptation when the number of samples available in the dataset was enough. Additionally, although both libraries performed reasonably well on the task, using deeper and pre-trained models on audio signals made a notable difference in the learning capacity of the models.

### 5.2. Facial Emotion Recognition Results

For the facial emotion recognizer, we adjusted the weights of the pre-trained STN on the AffectNet dataset to apply transfer-learning strategies. The trained STN for sentiment recognition on AffectNet reached an accuracy of 70.60%, as we can see in [60]. However, applying Feature-Extraction and max. voting on the posteriors, we required another model able to solve emotion recognition. Therefore, we trained the STN again with the same database using seven emotions, the same as RAVDESS except for the ‘Calm’ emotion. This second model reached an accuracy of 65.90% on the AffectNet database using the same parameters and evaluation strategy as in [60].

Although the task of sentiment recognition and emotion recognition seems related, we performed two experiments to evaluate if our hypothesis was true. In Table 2, we can see the difference in accuracy when we fine-tuned the model using pre-trained weights against training the network from scratch. The results showed that the weights learned with AffectNet were a suitable baseline to recognize emotions on RAVDESS compared with using random weights initialization. The first strategy achieved a significant improvement of 14.32% against the second.

After confirming this fact, we explored the two transfer learning strategies on the pre-trained STN on AffectNet, as we can see in Table 3. First, we applied Feature-Extraction from the posteriors, the fc50, and the flattened-810 layers. In this case, the top accuracy reached by all the combinations of the tested models was 53.85% when we used the 810-dimensional embeddings in a two layer bi-LSTM with 200 neurons and two attention layers. We can see that the sequential model also surpassed the accuracy reached by the max. voting strategy when there was no adaptation to the RAVDESS dataset.

When we adapted the model to the RAVDESS dataset, we can see that again, the 810-dimensional embeddings reached a top accuracy of 57.08%. Although this rate was higher than that obtained with the model without adaptation, the improvement did not fulfill the expectation created by the speech models that improved by 36.85 points after Fine-Tuning the base models.

**Figure 6:**
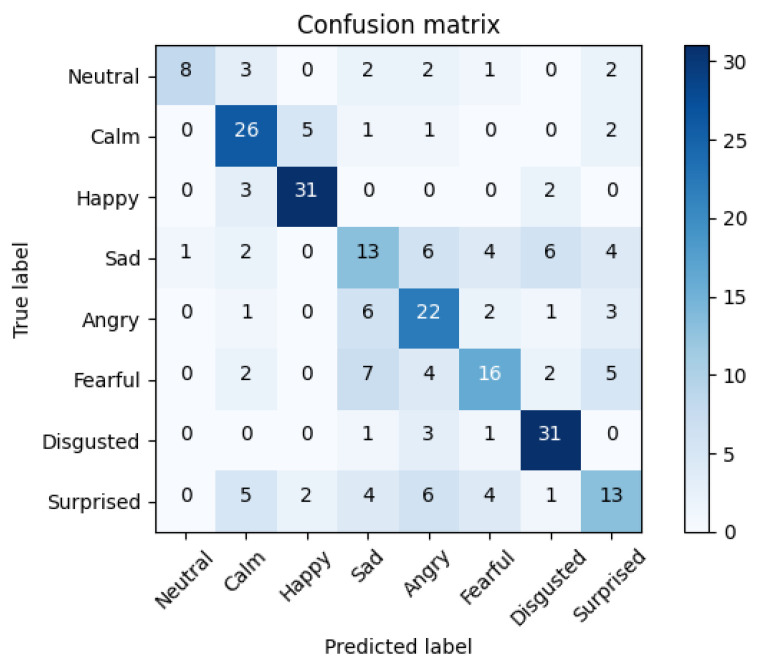
Average confusion matrix of the bi-LSTM with two layers of 300 neurons and two attention layers trained with the embeddings extracted from the flattened-810 of the fine-tuned STN. Accuracy of 57.08%. See Table 3.

To analyze the cause of the errors, we plotted the confusion matrix of the top experiment that reached an accuracy of 57.08%. In Figure 6, we can see the average confusion matrix.

The matrix shows that most errors were committed between the classes ‘Sad’ and ‘Fearful’, or ‘Angry’ and ‘Sad’. These results may indicate that the weights from AffectNet for valence/sentiment recognition did not adapt completely to the new task of emotion recognition, maintaining decisions typical from a valence recognizer. The possible causes could be the reduced size of the network, that may improve by introducing more layers; or the noise introduced by the frames of the videos. Regarding the noise problem, as for the training of the STN we needed to assign one label per frame, all the frames of the same video had to heritage the label of the parent; nevertheless, not all the images that conformed to the video represented the emotion of the ground truth in the same way, which seemed to introduce noise in the training process.

**Figure A1:**
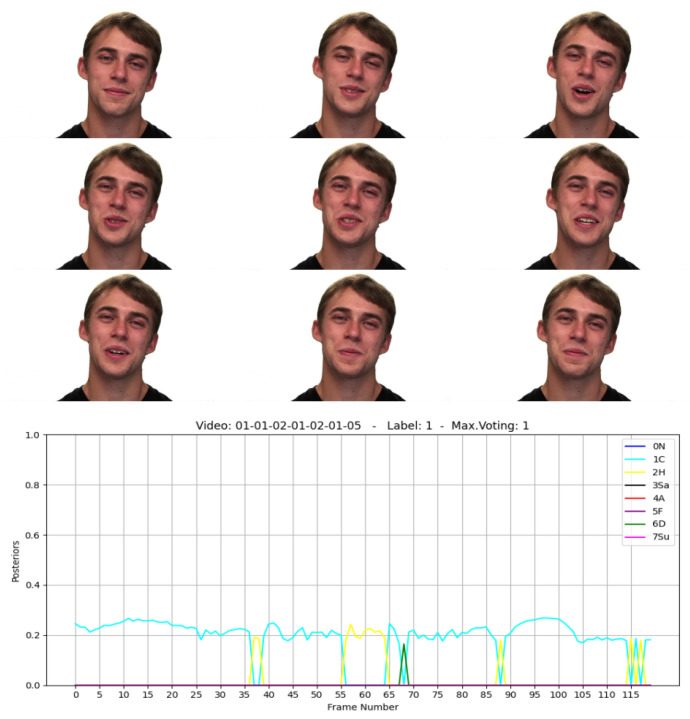
Example of frames from a video tagged as ‘Calm’ with some samples predicted as ‘Happy’. The whole video was correctly predicted as ‘Calm’.

**Figure A2:**
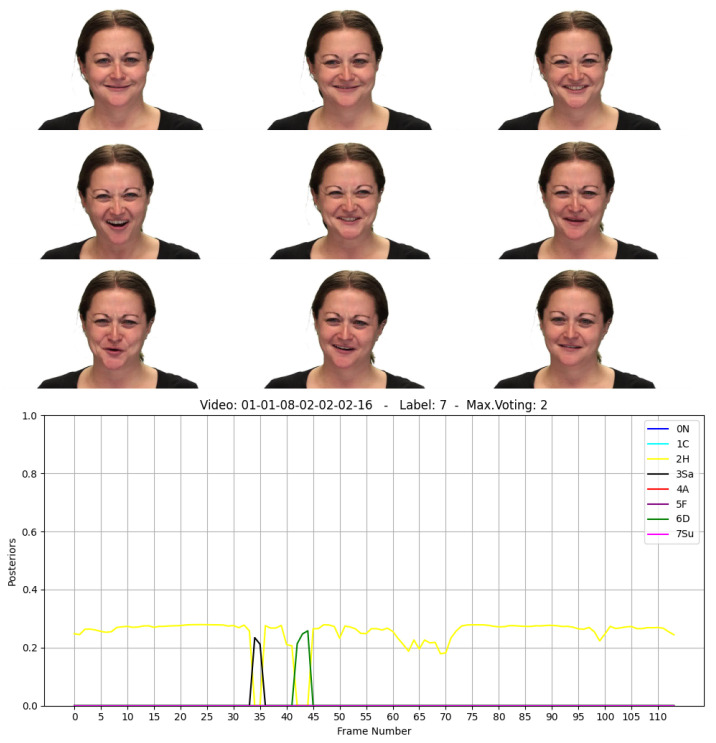
Example of frames from a video tagged as ‘Surprised’ incorrectly predicted as ‘Happy’. The whole video was incorrectly predicted as ‘Happy’.

**Figure A3:**
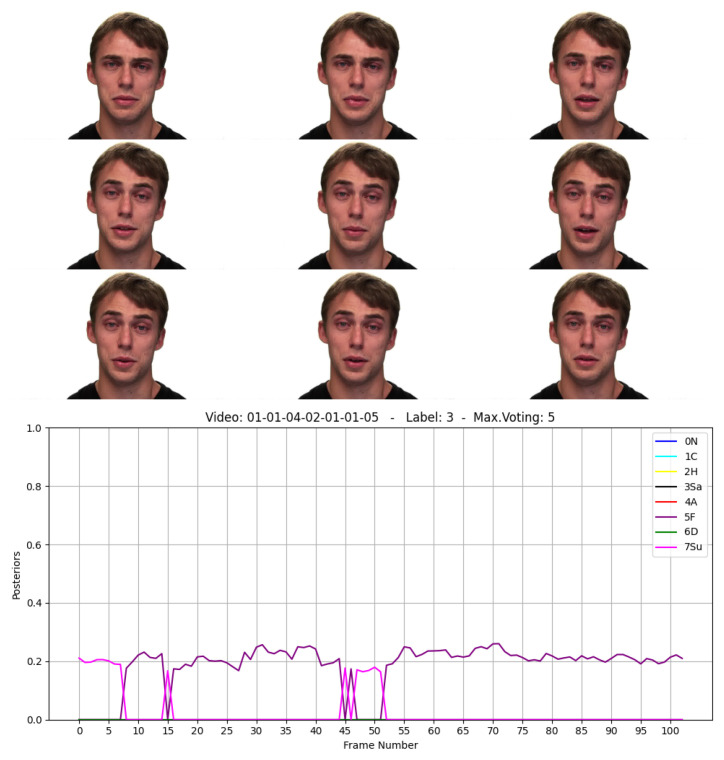
Example of frames from a video tagged as ‘Sad’ incorrectly predicted as ‘Fearful’. The whole video was incorrectly predicted as ‘Fearful’.

Figure A1–Figure A3 show some examples of what we have commented, extracted from several videos evaluated with the max. voting strategy and the 8-dimensional posteriors. In Figure A1, we represent some frames of a video labeled as ‘Calm’. As we can see, although the final prediction at the video level is correct (‘Calm’ or 1), in some moments the model returns ‘Happy’ or 2. In Figure A2, we show a video with assigned ground-truth as ‘Surprised’, but displaying what we could consider as ‘Happy’ in many frames, which was also the decision of the predictor. Figure A3 represents a more subtle error. Here, the STN mistakes the emotion ‘Sad’ with ‘Fearful’, which is a reasonable error since these emotions share some common patterns. Some possible solutions to this problem are the creation of a detector of ‘good’ frames, which may rely on a threshold over the posteriors of an emotion recognition model based on images to fine-tune the model only on the most probable classes; or training the model with the whole clip sequence in an end-to-end way.

### 5.3. Multimodal Fusion Results

**Figure 7:**
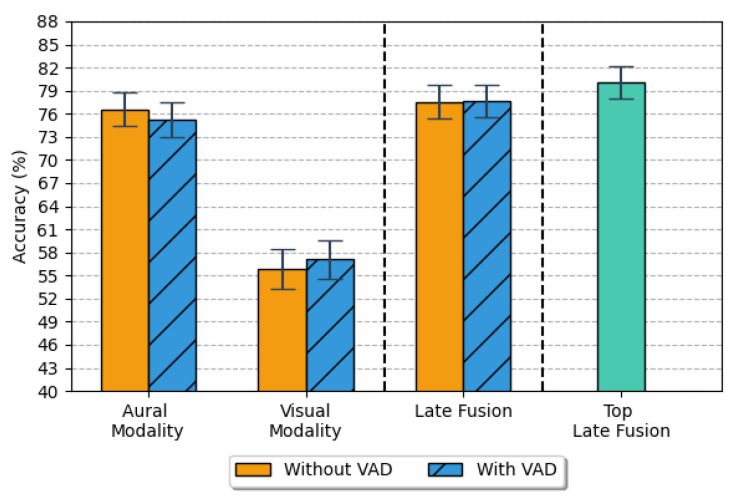
The top average accuracy of the 5-CV obtained for speech and visual modalities with a 95% confidence interval. In orange, the experiments with the original videos; in blue, the samples with speech; in green, the mix of the top modalities: the speech model without VAD and the visual model with VAD.

Although the results achieved by the visual modality were inferior to those reached by the speech system, we can see that the final accuracy obtained from the late fusion of the posteriors improved both of the single modalities, accomplishing the best fusion accuracy of 80.08% against the 57.08% of the visual modality and the 76.58% of the speech modality. Figure 7 shows the top accuracy reached by every single modality and the late fusion results with VAD, without VAD, and mixing the speech version without VAD and the visual version with VAD.

From the application of a nonparametric analysis, the Cochran’s Q test found that there were statistically significant differences in the proportions across the three top models of each modality (*p-value* < 0.001). Pairwise comparison using the McNemar’s test indicate at the overall 0.05 level that the late fusion model (M = 80, STD = 39.90) and the speech-based model (M = 77, STD = 42.30) were better at emotion recognition than the visual-based model (M = 77, STD = 42.30) with *p-value* < 0.001. Regarding the aural modality against the fusion version, from the tests, we can conclude that the differences are also significant between these two models with a *p-value* < 0.001.

**Figure 8:**
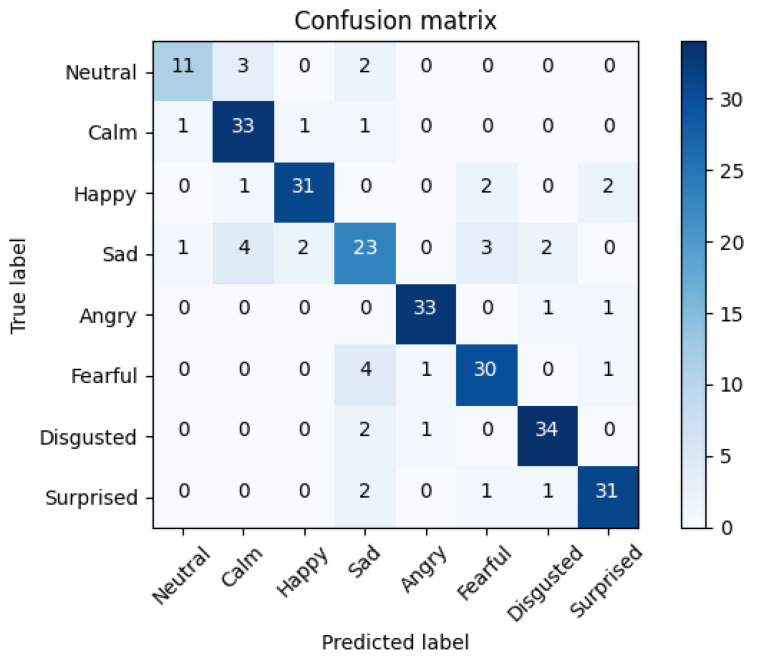
Average confusion matrix of the top late fusion strategy using a LinearSVC combining the top results of SER for the version without VAD and the FER for the version with VAD. Accuracy of 80.08%. See Table A3.

In Figure 8, we show the average confusion matrix of the late fusion strategy using the linear SVM with a C=0.001. Comparing the matrices of the visual and aural modalities, we can see how the combination of both modalities helps to correct deficiencies, especially for the visual modality, where emotions such as ‘Angry’, ‘Fearful’, or ‘Surprised’, which on the image-based model showed many errors, improved by adding the speech emotion recognizer to the fusion.

On the contrary, compared with the previous results of the speech-based model, the combination modified the errors and success distribution between classes, although both matrices are similar since the increase is not as notable as for the visual modality, as we can see comparing their mean accuracy.

As a conclusion, comparing the visual and aural matrices in terms of emotions, we can see that ‘Happy’ is more easily found by the visual model than by the aural model, as opposed to what happens with the ‘Angry’, ‘Sad’, ’Fearful’, or ‘Surprised’. These results suggest that ‘Happy’ has patterns more easily to differentiate visually (as smiling) from other emotions than ‘Angry’, ‘Sad’, or ‘Fearful’ that visually could share some similar characteristics more adequately modeled by the aural modality.

Regarding the use or not of the VAD, the graphs of Figure 7 shows that the effect of using the VAD strategy or not is marginal, probably because the first and last frames do not carry a relevant load of emotional information compared to the central frames.

### 5.4. Comparative Results with Related Approaches

One of the main problems in comparing the results on RAVDESS is the diversity of the setups that appear in the literature. The most reliable result for comparing our experiments is that developed by Issa et al. [41]. Here, the researchers developed a speech emotion recognizer and applied a 5-CV, obtaining an accuracy of 71.61%. Dissanayake et al. [80] used the last two participants in the validation and test sets, respectively, reaching an accuracy of 56.71% on the speech modality. With a variation of this setup, Pepino et al. [40] used as the test set only the last two participants and combined the ‘Calm’ and ‘Neutral’ emotions, passing from a problem with eight emotions to one with seven different classes. In these conditions, the top accuracy reached by their model was 77.5%, applying a global normalization.

With our audio-based model, we reached an accuracy of 76.58%, achieving a 4.97% absolute improvement relative to the work of Issa et al. [41], and an 8.47% improvement if we compare this result with our multimodal solution.

### 5.5. Limitations

Among the limitations of the proposed models, we have to comment that we used the RAVDESS dataset to train the final models. This dataset contains only North American speakers, which may reflect performance degradation with people from different nationalities. Furthermore, the age may induce erroneous predictions since the actors of the dataset were all of median age, between 20 and 50 years approximately. Thus, applying these models to babies or older people may reveal accuracy reduction due to the facial morphology in higher or lower age ranges.

The models also could show low performance under challenging lighting conditions or partial occlusions since the training images were collected in a laboratory environment with a white background, clear lighting conditions, and without fast movements of the participants. Regarding the sequential models, they could also introduce a small bias in the wild although on a minor scale since the max. voting and sequential strategies reported similar results. In conclusion, the temporal models seem not to be as relevant in performance degradation as discovering the most suitable frames or the recording conditions for the visual modality.

## 6. Conclusions

The automatic classification of emotions is a challenging task. Although there seem to exist similar patterns, there are still many variations between individuals, even although they are actors or from the same nationality, as with the RAVDESS database.

In this paper, we evaluated a multimodal system for emotion recognition that worked on the basis of speech and facial information.

Regarding the speech-based models, we demonstrated that the fine-tuned model using a conventional CNN from the PANNs framework outperformed other proposals with different networks, as AlexNet. The main reason for this improvement is probably because the CNN-14 was trained using audio samples against the AlexNet network that was trained with images.

Comparing our speech model with human perception, our model reached an increment of 9.58% points, demonstrating the robustness of the proposed procedure for this modality. Additionally, our proposal also outperformed the solution in Issa et al. [41] by 4.97%.

For the visual modality, we adapted a pre-trained STN for valence recognition to the emotion recognition task, reaching an accuracy of 57.08% when we used the 810-dimensional embeddings from the STN together with a bi-LSTM. Although results improved the Feature Extraction version, they did not increase as expected after adapting it to the database. This result reveals certain issues that will be corrected in the future for modeling the dynamic nature of the emotions represented in a video. This temporal structure inherent to video content makes that when it is divided into frames, the images lose their temporal meaning and single frames may not represent the emotion of the whole video, as we can see in Figure A2. We believe that this fact deteriorated the training of the networks since non-temporal models tried to learn from fragmented information giving the same weights to all the samples, but in videos, this fact could not apply. As a consequence, the training of the model was stuck in lower performances, as seemed to happen with the STN. This difficulty in the training of the STN model also affected the subsequent modules. For this reason, the difference in performance between the maximum voting algorithm and the bi-LSTM was minimum, as we can see in Table 3. Furthermore, in the confusion matrix of Figure 6 we see that the model kept the knowledge of the previous task, valence recognition, as it reflected most of its errors.

Despite the lower performance of the visual modality concerning the speech modality, the fusion of both sources let us achieve 80.08% accuracy in emotion classification.

From the experiments with the InaSpeech Voice Activity Detector, we have seen that removing the initial and final silences did not impact in a significant way the final results, so they could be removed without affecting the final performance, reducing the amount of information to process. More experiments with different VADs should be performed to check whether it is possible to train better models from samples where speech appears or with only silences.

As future lines, we plan to improve the visual model by changing its architecture and probably append it into a temporal model to learn information from the whole audio. Furthermore, we will continue with the study of different VADs to select those fragments with a higher emotional load that can help to distinguish emotions and remove noisy frames. Finally, we will test these strategies in new datasets and evaluate the model in real conditions.

## Figures and Tables

**Table 1:** Quantitative evaluation of the different strategies on speech emotion recognition. In bold, the best model.

TL Strategy	Inputs	Models	With VAD (InaSpeech)	Accuracy ± 95% CI
-	-	Human perception [18]	-	67.00
-	-	ZeroR	-	13.33 ± 2.06
Feature Extraction	Deep-Spectrum embs. from fc7 of AlexNet	SVC	No	43.32 ± 2.56
			Yes	45.80 ± 2.57
	PANNs embs. from CNN-14	SVC	No	39.73 ± 2.53
			Yes	37.22 ± 2.50
Fine Tuning	Mel spectrograms	AlexNet	No	60.72 ± 2.52
			Yes	61.67 ± 2.51
	Mel spectrograms	CNN-14	No	**76.58 ± 2.18**
			Yes	75.25 ± 2.23

**Table 2:** Evaluation of different initialization strategies for Fine-Tuning the STN on RAVDESS: random initialization vs. weights from pre-trained STN on AffectNet dataset.

Initialization Strategy	Inputs	Model	Accuracy ± 95% CI
Training from scratch	Frames and saliency maps	STN + max. voting	39.88 ± 2.53
Fine-Tuning AffectNet weigths	Frames and saliency map	STN + max. voting	54.20 ± 2.57

**Table 3:** Quantitative evaluation of the different strategies on the facial emotion recognizer. Results are given at the video level. All the results are reported on eight emotions except those that appear with (*), that are reported in seven emotions, collapsing the ‘Neutral’ and ‘Calm’ emotions. In bold, the best model.

TL Strategy	Inputs	Models	With VAD (InaSpeech)	Accuracy ± 95% CI
-	-	Human perception [18]	-	75.00
-	-	ZeroR	-	13.33 ± 2.06
Feature Extraction (from pre-trained STN on AffectNet)	posteriors (7 classes)	Max. voting	No	30.49 * ± 2.38
			Yes	30.35 * ± 2.37
		Sequential (bi-LSTM)	No	38.87 ± 2.52
			Yes	39.75 ± 2.53
	fc50	Sequential (bi-LSTM)	No	50.40 ± 2.58
			Yes	48.77 ± 2.58
	flatten-810	Sequential (bi-LSTM)	No	53.85 ± 2.57
			Yes	51.70 ± 2.58
Fine-Tuning on RAVDESS	posteriors (8 classes)	Max. voting	No	54.20 ± 2.56
			Yes	55.07 ± 2.56
		Sequential (bi-LSTM)	No	55.82 ± 2.56
			Yes	56.87 ± 2.56
	fc50	Sequential (bi-LSTM)	No	46.48 ± 2.58
			Yes	46.13 ± 2.57
	flatten-810	Sequential (bi-LSTM)	No	54.14 ± 2.57
			Yes	**57.08 ± 2.56**

## Data Availability

The RAVDESS database used in this paper is available under request from https://zenodo.org/record/1188976#.YTscC_wzY5k, accessed on 10 September 2021.

## Abbreviations

The following abbreviations are used in this manuscript:

FER	Facial Emotion Recognition
SER	Speech Emotion Recognition
RAVDESS	The Ryerson Audio-Visual Database of Emotional Speech and Song
ST	Spatial Transformer
CNN	Convolutional Neural Network
MTCNN	Multi-task Cascaded Convolutional Networks
Bi-LSTM	Bi-Directional Short-Term Memory networks
GAN	Generative Adversarial Networks
embs	embeddings
fc	fully-connected
SVC	Support Vector Machines/Classification
VAD	Voice Activity Detector
TL	Transfer-Learning
CI	Confidence Interval
CV	Cross-Validation

## Appendix A. Architecture Layers and Dimensions of the Spatial Transformer Network

In Table A1, we summarize the layers of the network used during the Fine-Tuning step on RAVDESS dataset. It has a final fully connected of size 8, one neuron per emotion predicted.

**Table A1:** Layers of the STN with their parameters.

Branches of the Model	Input Layer	Output Size	Filter Size/Stride	Depth
Input	Input Image	48 × 48 × 1	-	-
Localization Network	Convolution-2D	42 × 42 × 8	7 × 7/1	8
	MaxPooling-2D	21 × 21 × 8	2 × 2/2	-
	Relu	21 × 21 × 8	-	-
	Convlution-2D	17 × 17 × 10	5 × 5/1	10
	MaxPooling-2D	8 × 8 × 10	2 × 2/2	-
	Relu	8 × 8 × 10	-	-
	Fully-Connected	640	-	-
	Fully-Connected	32	-	-
	Relu	32	-	-
	Fully-Connected (θ)	6	-	-
Input	Transformed Image	48 × 48 × 1	-	-
Simple-CNN	Convolution-2D	46 × 46 × 10	3 × 3/1	10
	Relu	46 × 46 × 10	-	-
	Convolution-2D	44 × 44 × 10	3 × 3/1	10
	MaxPooling-2D	22 × 22 × 10	2 × 2/2	-
	Relu	22 × 22 × 10	-	-
	Convolution-2D	20 × 20 × 10	3 × 3/1	10
	Relu	20 × 20 × 10	-	-
	Convolution-2D	18 × 18 × 10	3 × 3/1	10
	Batch Normalization	18 × 18 × 10	-	-
	MaxPooling-2D	9 × 9 × 10	2 × 2/2	-
	Relu	9 × 9 × 10	-	-
	Dropout (*p* = 0.5)	9 × 9 × 10	-	-
	Flatten	810	-	-
	Fully-Connected	50	-	-
	Relu	50	-	-
	Fully-Connected	8	-	-

## Appendix B. Top bi-LSTM Models with Attention Mechanism

In Table A2, we show the architectures of the top sequential models used, whose results were reported in Table 3.

**Table A2:** Table with model architectures associated to the rates reported in Table 3.

TL Strategy	Inputs	Models	With VAD (InaSpeech)	Model Architecture
-	-	Human perception	-	-
-	-	ZeroR	-	-
Feature Extraction (from pre-trained STN onAffectNet)	posteriors (7 classes)	Max. voting	No	-
			Yes	-
		Sequential (bi-LSTM)	No	1 layer bi-LSTM with 150 neurons +1 attention layer
			Yes	1 layer bi-LSTM with 150 neurons +1 attention layer
	fc50	Sequential (bi-LSTM)	No	2 layers bi-LSTM with 50 neurons +1 attention layer
			Yes	1 layer bi-LSTM with 150 neurons +1 attention layer
	flatten-810	Sequential (bi-LSTM)	No	2 layer bi-LSTM with 200 neurons +2 attention layers
			Yes	1 layer bi-LSTM with 150 neurons +1 attention layer
Fine-Tuning on RAVDESS	posteriors (8 classes)	Max. voting	No	-
			Yes	-
		Sequential (bi-LSTM)	No	2 layer bi-LSTM with 50 neurons +2 attention layers
			Yes	1 layer bi-LSTM with 25 neurons +1 attention layer
	fc50	Sequential (bi-LSTM)	No	1 layer bi-LSTM with 150 neurons +1 attention layer
			Yes	2 layer bi-LSTM with 150 neurons +2 attention layers
	flatten-810	Sequential (bi-LSTM)	No	2 layer bi-LSTM with 150 neurons +2 attention layers
			Yes	2 layer bi-LSTM with 300 neurons +2 attention layers

## Appendix C. Examples of Frames Extracted from the RAVDESS Videos

In this appendix, we show some incorrectly classified samples of the STN model of accuracy of 54.20% reported in Table 3 in which we combine the posteriors applying a max. voting strategy. These examples help to understand the challenge of predicting emotions from videos using single frames. We also plot the posteriors of the model per emotion during the whole video.

## Appendix D. Evaluation of the Fusion Models

In Table A3, we can see the three models tested for applying the fusion strategy that are a Logistic Regression, a SVM and a k-NN with a max. voting strategy. On the second column, we can see the tuned hyperparameters on each model, maintaining for the other parameters the default values implemented in the sklearn library [71]. Finally, the accuracy is obtained from different combinations of the top models of the visual and aural modalities. The VAD version is the result of combine the outputs of the CNN-14 for SER with accuracy of 75.25% and the top bi-LSTM with attention for FER with accuracy of 57.08%. The version without VAD combines the outputs of the SER model with accuracy of 76.58% and the FER with 57.08%; and the mixed version fused the outputs of the SER model with accuracy of 76.58% with the FER model with accuracy of 57.08%.

**Table A3:** Tested models for applying fusion strategy on the combined posteriors of the top models for each modality. In bold, the best models of each type in terms of the accuracy.

Fusion Model	Hyper Parameters	Accuracy with VAD	Accuracy without VAD	Accuracy withVAD FER and withouth VAD SER
Logistic Regression	C=0.1	71.6	68.18	70.72
	C=1.0	71.93	69.27	71.13
	C=10	72.76	69.47	71.47
	$C=10^{2}$	76.62	73.50	75.68
	$C=10^{3}$	77.78	76.98	79.35
	$C=10^{4}$	77.33	77.33	79.63
	$C=10^{5}$	77.55	77.68	**79.70**
	$C=10^{6}$	77.55	77.78	79.63
SVM	kernel = ‘linear’; $C=10^{-4}$	77.4	77.37	79.58
	kernel = ‘linear’; $C=10^{-3}$	77.65	77.80	**80.08**
	kernel = ‘linear’; $C=10^{-2}$	77.70	77.20	79.20
	kernel = ‘linear’; C=0.1	77.70	76.17	78.77
	kernel = ‘linear’; C=1	77.83	76.68	78.77
	kernel = ‘linear’; C=10	77.77	76.82	78.77
	kernel = ‘rbf’; C=0.1	60.03	59.62	62.47
	kernel = ‘rbf’; C=1	66.95	61.57	63.57
	kernel = ‘rbf’; C=10	70.10	64.30	66.48
	kernel = ‘rbf’; $C=10^{2}$	70.10	64.33	66.57
k-NN	k = 10	60.32	59.30	**60.33**
	k = 20	58.95	57.15	59.07
	k = 30	57.63	57.78	59.17
	k = 40	56.78	57.55	59.01
	k = 50	55.90	57.00	58.65
